# Spectrophotometric Methods Based on Charge Transfer Complexation Reactions for the Determination of Saxagliptin in Bulk and Pharmaceutical Preparation

**Published:** 2012-09

**Authors:** Ramzia I. El-Bagary, Ehab F. Elkady, Bassam M. Ayoub

**Affiliations:** *Department of Pharmaceutical Chemistry, Faculty of Pharmacy, Cairo University, Kasr El-Aini St., Cairo 11562, Egypt*

**Keywords:** saxagliptin, spectrophotometry, charge transfer reactions, pharmaceutical preparation

## Abstract

Simple, accurate and precise spectrophotometric methods have been developed for the determination of saxagliptin in bulk and dosage forms. The proposed methods are based on the charge transfer complexes of saxagliptin with 2,3-dichloro-5,6-dicyano-1,4-benzoquinone (DDQ) and 7,7,8,8-tetracyanoquinodimethane (TCNQ). All the variables were studied to optimize the reactions’ conditions. Beer’s law was obeyed in the concentration ranges of 50-300 μg/ml and 10-110 μg/ml with DDQ and TCNQ, respectively. The developed methods were validated and proved to be precise and accurate for the quality control of the saxagliptinin its pharmaceutical dosage form.

## INTRODUCTION

**Figure 1:**
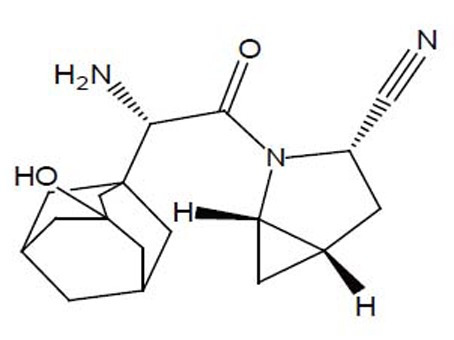
Chemical structure of saxagliptin.

Saxagliptin (SXG),(1S,3S,5S)-2-[(2S)-2-amino-2-(3-hydroxy-1-adamantyl)acetyl]-2-azabicyclo [3.1.0] hexane-3-carbonitrile (Fig. 1) is a new oral hypoglycemic drug of the new dipeptidyl peptidase-4 (DPP-4) inhibitor class of drugs (1). Saxagliptin is recently approved for the treatment of type 2 diabetes mellitus (2). DPP-4 inhibitors represent a new therapeutic approach to the treatment of type 2 diabetes that functions to stimulate glucose-dependent insulin release and reduce glucagons levels. This is done through inhibition of the inactivation of incretins, particularly glucagon-like peptide-1 (GLP-1) and gastric inhibitory polypeptide (GIP), thereby improving glycemic control (3). Literature survey reveals that the drug has been estimated by only one LC-MS/MS (4) and another spectrophotometric method in which saxagliptin was estimated at 208 nm in methanol. Linearity range was found to be 5-40 μg/ml. The LOD and LOQ were found to be 0.06 μg/ml and 0.18 μg/ml respectively (5).

The aim of the work is to present the first charge transfer complexation methods for the determination of SXG in bulk and pharmaceutical formulations. Charge transfer reactions have been widely used for the determination of electron donating compounds through interaction with π-acceptors. Among the electron acceptors mostly used in literature are 2,3-dichloro-5,6-dicyano-1,4-benzoquinone (DDQ) (6-9) and 7,7,8,8-tetracyanoquinodimethane (TCNQ) (10-13).

## EXPERIMENTAL

### Instrumentation

A Jenway 6800 double beam ultraviolet/visible spectrometer connected to an IBM compatible computer with 1-cm quartz cell and supported with Jenway flight deck software was used.

### Reagents and reference samples

DDQ and TCNQ were supplied from Sigma Aldrich Chemie GmbH (Steinheim, Germany). Freshly prepared solutions were prepared (DDQ solution 16.2 × 10^-3^ M in methanol), (TCNQ solution 5.4 × 10^-3^ Min methanol). Pharmaceutical grade SXG certified to contain 99.85% and Onglyza^®^ tablets nominally containing 5 mg of SXG per tablet were kindly supplied by Bristol-Myers Squibb/AstraZeneca EEIG (United Kingdom). Standard stock solutions of SXG (1 mg/ml) was prepared by dissolving 100 mg of SXG in methanol in a 100 ml volumetric flask and completing to volume with methanol. All the solvents used were of analytical grade.

### General procedures and calibration graphs

**Method using DDQ.** Aliquots of SXG containing (0.5-3 mg) were transferred into a set of 10 ml volumetric flasks, treated with 1 ml DDQ solution and allowed to stand for 40 min at room temperature (20-25°C) and diluted to volume with methanol. The absorbance was measured at 461 nm against reagent blank.

**Method using TCNQ.** Aliquots of SXG containing (0.1-1.1 mg) were transferred into a set of 10 ml volumetric flasks, treated with 1 ml TCNQ solution and allowed to stand for 30 min at room temperature (20-25°C) and diluted to volume with methanol. The absorbance was measured at 838 nm against reagent blank.

### Procedure for the assay of the tablets

Twenty tablets of were weighed and the coats were removed by carefully rubbing with a clean tissue wetted with using methanol. An accurately weighed amount of the finely powdered tablets equivalent to 100 mg was dissolved in methanol in a 100 ml volumetric flask, sonicated for 30 minutes and completed to volume with methanol. The solution was then filtered, followed by serial dilution to required concentrations. The procedure was continued as mentioned under general procedures and calibration graphs.

### Effect of the amount of the reagent

Aliquots of SXG (2.7 × 10^-3^ M) stock solutions were introduced into a set of 10 ml volumetric flasks. Different aliquots of (DDQ and TCNQ) were added to each flask to obtain different drug/ reagent molar ratios in an increasing order, and then the procedure was continued as mentioned under general procedures and calibration graphs.

### Stoichiometric relationship

Job’s method of continuous variation was employed, between standard solutions of 2.7 × 10^-3^ M of SXG) with the two reagents (DDQ with concentration 2.7 × 10^-3^ M and TCNQ with concentration 2.7 × 10^-3^ M). A series of solutions was prepared in which the total volume of the drug and the reagent was kept at 5 ml. The method was continued as mentioned under the general procedures for the calibration graphs.

## RESULTS AND DISCUSSION

### Formation of the charge transfer complexes

The charge transfer reagents applied in this work are DDQ and TCNQ. DDQ is an electron deficient molecule due to the electron withdrawing effect of the two cyano and the two chloro groups. The reaction between DDQ as a π-acceptor with several drugs (6-9) to give a complex which dissociates in polar solvents to a highly colored radical anion has been reported. Likewise, TCNQ is a well known π-acceptor (10-13); this character is derived from the high electron affinity of polyene system conferred by the electron withdrawing effect of the four cyano groups and also from the planarity and high symmetry of TCNQ.

### Optimization of the reactions' conditions

**Figure 2:**
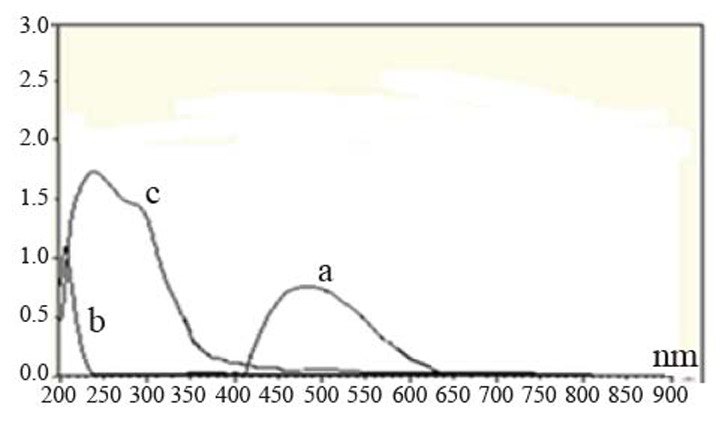
A bsorption s pectra of t he c olored p roduct of s axagliptin (2.7 × 10^-3^ M) and DDQ (16.2 × 10^-3^ M) in methanol (a), saxagliptin (2.7 × 10^-3^ M) in methanol (b) and DDQ (16.2 × 10^-3^ M) in methanol (c).

**Figure 3:**
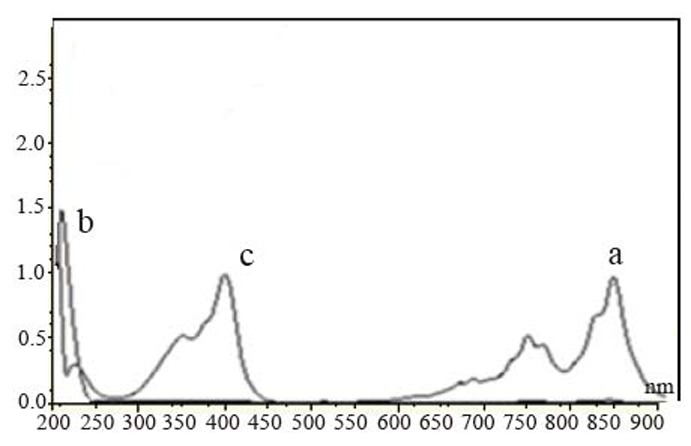
Absorption spectra of the colored product of TCNQ (5.4 × 10^-3^ M) with saxagliptin (2.7 × 10^-3^ M) in methanol (a), saxagliptin (2.7 × 10^-3^ M) in methanol (b), TCNQ (5.4 × 10^-3^ M) in methanol (c).

Different parameters affecting the reactions such as amount of the reagent, reaction time and stability of the color have been investigated. λ_max_ of measurements of SXG with the two reagents are shown in Table 1. The absorption spectra of the reaction products of SXG with the two reagents are shown in Figures 2-3.

**Table 1:** Results obtained by the proposed methods for the determination of saxagliptin using DDQ and TCNQ

Item	DDQ method	TCNQ method

Solvent	Methanol	Methanol
Time of reaction	40 minutes	30 minutes
Stability of the color	50 minutes	60 minutes
λ_max_ of measurements	461nm	838 nm
Obedience of Beer’s law	50-300 μg/ml	10-110 μg/ml
Regression equation	A_486_ = 0.0041 × Conc _(μg/ml)_ – 0.0457	A_838_ = 0.0107 × Conc_(μg/ml)_ + 0.0643
Regression coefficient (r^2^)	0.9999	0.9996
LOD μg/ml	2.53	2.79
LOQ μg/ml	7.68	8.46
S_b_	5.7 × 10^-5^	1.2 ×10^-4^
S_a_	1.4 × 10^-2^	9.9 × 10^-3^
Confidence limit of the slope	0.0041 ± 3.36 × 10^-2^	0.0107 ± 0.1 ×10^-3^
Confidence limit of the intercept	-0.0457 ± 2.6 × 10^-6^	0.0643 ± 7.7 × 10^-6^
Standard error of the estimation	0.012	9.9 ×10^-3^
Results		
Drug in bulk	99.90 ± 0.80	100.03 ± 1.32
Drug in dosage form	99.61 ± 0.63	100.17 ±1.13
Drug added	99.80 ± 1.16	100.47 ± 1.42

**Figure 4:**
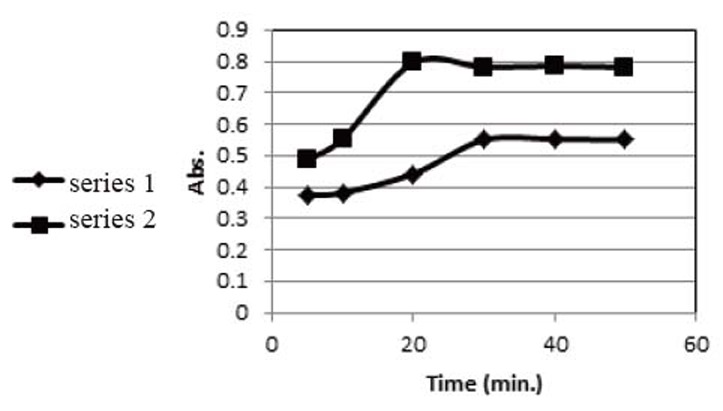
Effect of the reaction time in the determination of saxagliptin with DDQ (series 1) and TCNQ (series 2).

**Rate of complex formation.** A study of the effect of time revealed that the maximum color intensity was attained at least after 30 min with DDQ method and at least after 20 min with TCNQ method as shown in Figure 4.

**Figure 5:**
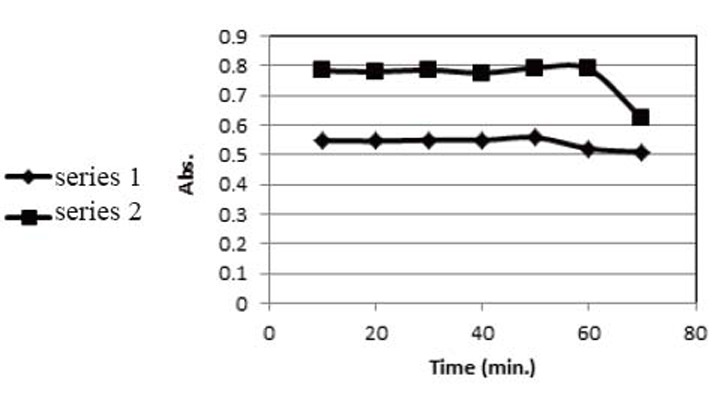
Effect of stability of the formed charge transfer complex in the determination of saxagliptin with DDQ (series 1) and TCNQ (series 2).

**Stability of the formed complex.** A study of the stability of the formed complex revealed that the reaction product remained stable for further 50 min with DDQ method and for further 60 min with TCNQ method as shown in Figure 5.

**Figure 6:**
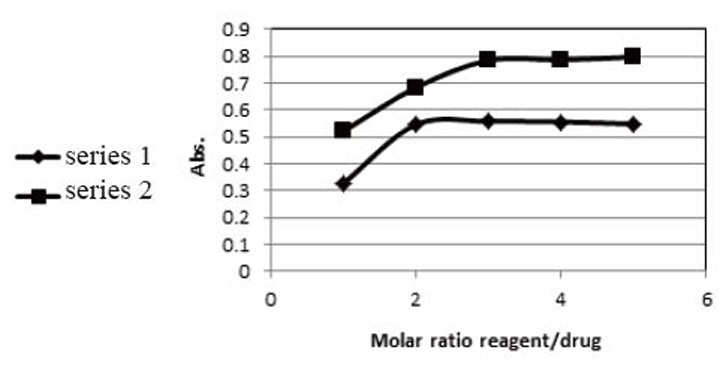
Effect of the amount of the reagent in the determination of saxagliptin with DDQ (series 1) and TCNQ (series 2).

**Effect of the amount of reagent.** Study of the amount of the reagent revealed that the amount of the reagent was optimized to be four times the amount of SXG in the charge transfer reaction with both DDQ and TCNQ as shown in Figure 6.

**Effect of temperature.** Study of the effect of temperature revealed that the reaction was optimized at room temperature in the charge transfer reaction with both DDQ and TCNQ.

### Stoichiometric relationship

**Figure 7:**
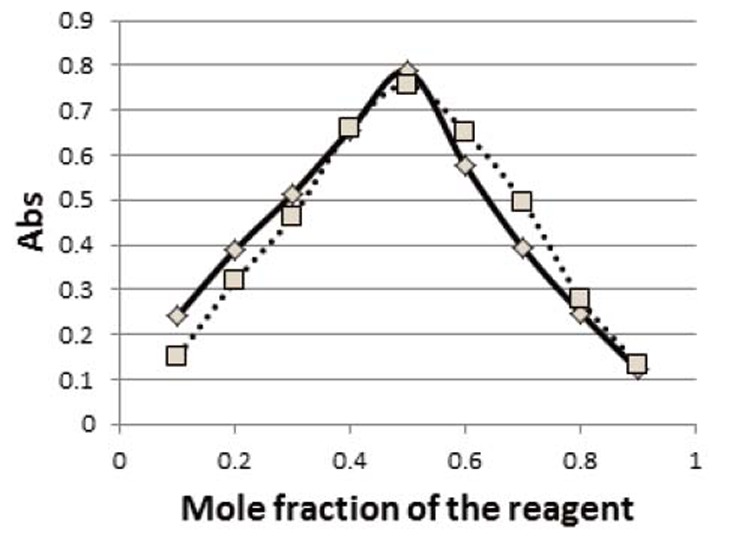
Continuous variation plot of the reaction products of saxagliptin with DDQ (—) and TCNQ (----).

Job’s method of continuous variation revealed a molar ratio of of (1:1) drug: reagent was obtained with the two reagents as shown in Figure 7. This may be attributed to the availability of one center as an electron donating group which is the primary amine while the amide nitrogen and cyano nitrogen are not electron donors.

### Quantification, accuracy and precision

Standard calibration curves were prepared by separately preparing series of different concentrations of SXG and applying the suggested procedures with DDQ and TCNQ. Beer’s law was valid within microgram concentration range of SXG (Table 1). The linearity of the calibration curves were validated by the high value of correlation coefficients. The analytical data of the calibration curves including standard deviations for the slope and intercept (S_b_, S_a_) are summarized in Table 1. The regression equations of these calibration graphs were utilized for determination of concentrations of the cited drug in bulk and tablets. The results obtained were of good accuracy and precision. The applicability of the procedures for estimation of tablets was validated using standard addition technique as a check for accuracy (Table 1).

### Statistical analysis

A statistical analysis of the results obtained by the proposed methods for the determination of SXG was carried out by “SPSS statistical package version 11”. The significant difference between groups including the reference method were tested by one way ANOVA (F-test) at *p*=0.05 as shown in Table 2. The test ascertained that there was no significant difference among the methods.

**Table 2:** Statistical comparison between the results of the proposed spectrophotometric methods and the reference method for the determination of saxagliptin

Statistical Term	Reference Method^b^	DDQ Method	TCNQ Method

Mean	100.20	99.90	100.03
S.D. ±	1.10	0.80	1.32
S.E. ±	0.49	0.36	0.59
%RSD	1.10	0.80	1.32
n	5	5	5
V	1.21	0.64	1.74
t (a2.306)		0.49	0.22

a Figures in parentheses are the theoretical t and F values at (*p*=0.05). No significant difference between groups of sitagliptin by using one way ANOVA with F equals 0.10 and p equals 0.91;

b Reference method: aliquots of standard solutions in distilled water containing 5-40 μg/ml were measured at 208 nm using water as a blank (4).

## CONCLUSION

The proposed spectrophotometric methods have the advantages of simplicity, precision, accuracy and convenience for the quantitation of SXG. Hence, the proposed methods can be used for the quality control of the cited drug and can be extended for routine analysis of SXG in bulk and dosage forms.
